# Signal enhancement ratio of CE-MRI: a potential biomarker of survival after hepatic arterial infusion chemotherapy in biliary tract cancers

**DOI:** 10.1186/s13244-022-01188-6

**Published:** 2022-03-14

**Authors:** Kanglian Zheng, Shijie Fu, Boyu Leng, Yong Cui, Renjie Yang, Guang Cao, Liang Xu, Wen-Qing Li, Ying Li, Xu Zhu, Song Gao, Peng Liu, Xiaodong Wang

**Affiliations:** 1 Key Laboratory of Carcinogenesis and Translational Research (Ministry of Education), Department of Interventional Therapy, Peking University Cancer Hospital & Institute, 52 Fucheng Road, Haidian District, Beijing, China; 2 Hebei North University, 11 Zuanshi South Road, Gaoxin District, Zhangjiakou, Hebei China; 3 Key Laboratory of Carcinogenesis and Translational Research (Ministry of Education), Department of Radiology, Peking University Cancer Hospital & Institute, 52 Fucheng Road, Haidian District, Beijing, China; 4 Key Laboratory of Carcinogenesis and Translational Research (Ministry of Education/Beijing), Department of Cancer Epidemiology, Peking University Cancer Hospital & Institute, 52 Fucheng Road, Haidian District, Beijing, China

**Keywords:** Biliary tract cancers, Contrast-enhanced MRI, Hepatic arterial infusion chemotherapy, Survival, Biomarker

## Abstract

**Background:**

The association of contrast-enhanced MRI (CE-MRI) and the overall survival (OS) of biliary tract cancers (BTC) is ambiguous. Thus, the aim of this study is to evaluate the value of signal enhancement ratio (SER) and its early change in CE-MRI as biomarkers of survival after hepatic arterial infusion chemotherapy (HAIC) in BTC.

**Results:**

One hundred and two BTC patients treated via HAIC with 3cir-OFF regimen between January 2011 and June 2020 were enrolled in this retrospective study. The median progression-free survival (PFS) and OS were 9.8 months [range 1.5–83.3 months, 95% confidence interval (CI) 7.789–11.811] and 14.2 months (range 1.8–83.3 months, 95% CI: 11.106–17.294), respectively. The cutoff value of SER before HAIC (SER_0_) was 1.04, and both median PFS and OS in the SER_0_ ≥ 1.04 group were longer than in the SER_0_ < 1.04 group (median PFS: 10.5 vs. 8.5 months, *p* = 0.027; median OS: 23.9 vs. 12.3 months, *p* < 0.001). The median OS in the ΔSER > 0 group was longer than in the ΔSER < 0 group (17.3 versus 12.8 months, *p* = 0.029 (ΔSER means the change of SER after two cycles of HAIC). Multivariate analysis showed SER_0_ (*p* = 0.029) and HAIC treatment cycle (*p* = 0.002) were independent predictors of longer survival.

**Conclusions:**

SER in CE-MRI before HAIC (SER_0_) is a potential biomarker for the prediction of survival after HAIC in advanced BTC.

## Key points

- SER of CE-MRI is a biomarker of survival for BTC after HAIC.
- ΔSER is also related to longer survival after HAIC in advanced BTC, but not an independent predictor of survival.

## Background

Biliary tract cancers (BTC) are malignancies arising from the biliary tracts with poor prognosis and are classified as gallbladder cancer (GBC), intrahepatic cholangiocarcinoma (iCCA), perihilar cholangiocarcinoma (pCCA), and distal cholangiocarcinoma (dCCA), based on anatomy. In the past decade, the incidence of BTC has increased worldwide, especially in China and southeast Asia [1–3].

Most BTC patients are diagnosed at the advanced stage and therefore are not suitable for surgery. Although systemic chemotherapy is currently the standard first-line treatment for BTC, results of randomized controlled trials have reported that the overall survival (OS) is usually less than one year [4, 5]. Hepatic arterial infusion chemotherapy (HAIC) has been proved to be a good alternative treatment in advanced iCCA [6]. HAIC with oxaliplatin and 5-fluorouracil has also been proved to have survival benefits with the median OS of 20.5 months for advanced pCCA [7] and 13.5 months for advanced GBC [8]. However, the prediction of survival for BTC remains a challenge.

In recent years, survival prediction using contrast-enhanced MRI (CE-MRI) has been proved to have value in hepatobiliary malignancy. In 2016, Fujita et al. found that the signal intensity (SI) of hepatocellular carcinoma (HCC) in the hepatobiliary phase (HBP) of CE-MRI before HAIC was correlated with prognosis in patients treated by HAIC [9]. In the same year, the relationship between the SI of mass-forming iCCA in HBP of CE-MRI and survival time was also proved [10]. Thus, the signal enhancement ratio (SER), one of the quantified indicators of CE-MRI, may have value for survival prediction for liver tumors and BTC.

Therefore, we retrospectively enrolled more than 100 patients with BTC treated by HAIC in our center in past consecutive 10 years to explore whether SER in CE-MRI could be a potential biomarker of survival after HAIC treatment in advanced BTC.

## Methods

### Patients

This retrospective study was supported by the institutional review board, and the requirement for informed consent was waived. All patients with BTC treated in our center from January 2011 to June 2020 were reviewed.

The eligibility criteria were as follows: (1) diagnosed as BTC by histopathology and cytopathology, including GBC, iCCA, and pCCA; (2) inoperable as confirmed by hepatobiliary surgeons and radiologists; (3) treated via HAIC with 3cir-OFF for at least two cycles; (4) received abdominal CE-MRI before the initiation of HAIC and after 2 cycles of HAIC; (5) at least one measurable lesion in CE-MRI images before the initiation of HAIC; (6) 18–80 years old; (7) Child–Pugh A/B; (8) Eastern Cooperative Oncology Group (ECOG) performance status (PS) ≤ 2; (9) tumor burden in the liver < 70% of the total liver volume; (10) adequate bone marrow function (white blood cell count ≥ 3.5 × 10^9^/L, absolute neutrophil count ≥ 1.5 × 10^9^/L, and platelet count > 75 × 10^9^/L); (11) adequate liver and renal function [alanine transaminase and aspartate transaminase ≤ 5 × upper limit of normal (ULN), total bilirubin (TBiL) < 5 × ULN, and serum creatinine < 2.0 mg/dL]; and (12) international normalized ratio ≤ 1.5.

The exclusion criteria were as follows: (1) coexistent or synchronous malignancies; (2) treated via HAIC combined with other treatment methods, such as systemic chemotherapy, targeted therapy, and trans-arterial chemoembolization (TACE); (3) a follow-up period < 6 months.

### Procedures and treatments

CE-MRI was performed on each patient within one month before the initiation of HAIC and after 2 cycles of HAIC. The images were assessed by two experienced radiologists (11 and 16 years of experience in abdominal imaging), and a consensus was reached. Complete blood count, blood biochemistry, blood coagulation, carcinoembryonic antigen (CEA), and carbohydrate antigen 19-9 (CA 19-9) were measured before HAIC.

A percutaneously implanted port-catheter system using fixed-catheter-tip technology, as depicted in published studies [11–14], was used for HAIC. The HAIC regimen was 3cir-OFF, which was composed of oxaliplatin (40 mg/m^2^ for 2 h), 5-flourouracil (800 mg/m^2^ for 22 h), and folinic acid (200 mg/m^2^) on days 1–3, every 3 or 4 weeks. The folinic acid was injected intravenously for 2 h from the beginning of 5-fluorouracil infusion on each day.

### Response evaluation and follow-up

The response was evaluated according to the *New response evaluation criteria in solid tumors: Revised RECIST guideline (version 1.1)* (RECIST 1.1) [15]. The follow-up processes, including abdominal CE-MRI scan, laboratory examination, and physical examination, were performed on every patient after two cycles of HAIC or every three months until the tumor progressed, or the patient died. OS was defined as the date from the initiation of HAIC to death, and progression-free survival (PFS) was defined as the date from the initiation of HAIC to tumor progression or death, whichever occurred first.

### CE-MRI imaging technique

All CE-MRI scans were performed with three MRI machines (machine 1: 3.0 T, Discovery MR750, GE, USA; machine 2: 1.5 T, Optima MR360, GE, USA; machine 3: 1.5 T, Aera, SIEMENS, Germany) with an eight-channel phased-array body coil. The contrast agent Gd-DTPA (Magnevist, Bayer Berlin, Germany) was injected intravenously (0.2 mL/kg, 2 mL/s), followed by a flush with 20 mL of saline solution using a power injector. The parameters of the contrast-enhanced T1-weighted image sequence were as follows: (1) machine 1: repetition time (TR), 4.01 ms; echo time (TE), 1.71 ms; matrix, 512 × 512; slice thickness, 5.0 mm; slice gap, 2.5 mm; flip angle, 10°; number of excitations (NEX), 0+. (2) machine 2: TR, 3.25 ms; TE, 1.43 ms; matrix, 256 × 256; slice thickness, 5.0 mm; slice gap, 2.5 mm; flip angle, 15°; NEX, 0+. (3) machine 3: TR, 4.50 ms; TE, 2.21 ms; matrix, 240 × 320; slice thickness, 3.5 mm; flip angle, 15°; NEX, 1.

### Image analysis

**Fig. 1:**
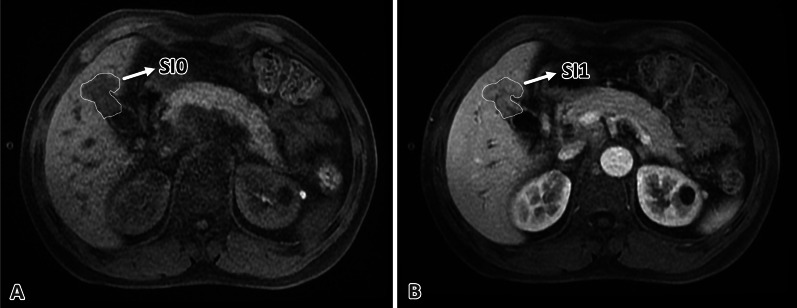
Drawing of the ROI in CE-MRI images.** A** Example of ROI drawing in the unenhanced phase image.** B** Example of ROI drawing in the portal vein phase image. SI, signal intensity; CE-MRI, contrast-enhanced MRI

The SI of the tumor was measured in a ROI, which included the substantial components as possible, in the longest axial image of the tumor in unenhanced and portal vein phase (Fig. 1). For patients with more than 2 lesions in the liver, two target lesions were selected based on RECIST 1.1, and the SI of the tumor was defined as the mean SI of the two lesions. The SER was calculated with the equation (SI_1_ – SI_0_)/SI_0_ [16], where SI_1_ was the SI of the tumor in portal vein phase and SI_0_ was the SI of the tumor in unenhanced phase. Then, ΔSER was calculated with the equation SER_0_ – SER_1_, where SER_0_ was the SER before the initiation of HAIC and SER_1_ was the SER after two cycles of HAIC.

### Statistical analysis

Continuous and categorical variables were presented as mean ± standard deviation and frequencies, respectively. Mann–Whitney *U* test, Kruskal–Wallis *H*, and Student’s *t*-test were used to analyze continuous variables. Chi-square tests and Fisher’s exact tests were used to analyze categorical variables. OS and PFS were analyzed using the Kaplan–Meier method and log-rank test. The receiver operating characteristic (ROC) curve was drawn; then, the Youden Index was calculated to obtain the cutoff value of SER_0_. Univariate and multivariate analyses were performed with the Cox proportional hazards regression method, and the factors with *p* < 0.10 in the univariate analysis were included in the multivariate analysis. A *p* < 0.05 was considered statistically significant. The Statistical Package for Social Sciences version 24.0 (IBM Corp., Armonk, NY, USA) was used for the statistical analysis.

## Results

### Patients

**Fig. 2:**
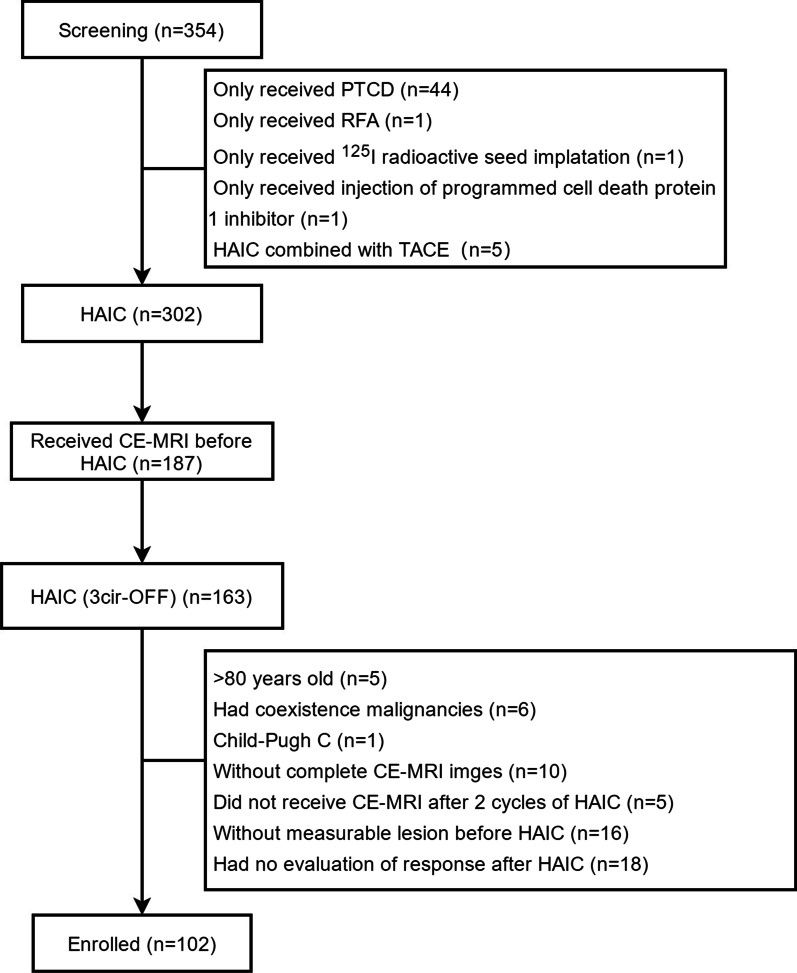
Patient flowchart. PTCD, percutaneous transhepatic cholangial drainage; RFA, radiofrequency ablation; HACI, hepatic arterial infusion chemotherapy; TACE, transarterial chemoembolization; CE-MRI, contrast-enhanced MRI

“Gallbladder cancer,” “biliary tract cancers,” “intrahepatic cholangiocarcinoma,” and “perihilar cholangiocarcinoma” were used as keywords to search in the Hospital Information System. Three hundred and fifty-four patients with BTC were treated in our center from January 2011 to June, 2020. According to the inclusion and exclusion criteria, 102 patients with BTC were enrolled in this study finally. The patients screen flowchart is shown in Fig. 2.

Out of these 102 patients (mean age, 60.38 ± 9.76 years; 56 males (54.9%)), 33 (32.4%) were diagnosed as iCCA, 14 (13.7%) as GBC, and 55 (53.9%) as pCCA. Twenty-five (24.5%) patients had recurrence after surgery or experienced progression after other treatments, and 71 (69.6%) suffered jaundice before the initiation of HAIC in which more than 70% of them contraindicated to systemic chemotherapy since their level of TBiL was over 2 × ULN. The characteristics of these patients are shown in Table 1.

**Table 1:** Univariate analysis of patients’ baseline characteristics and treatment factors on survival

Characteristics	Number of patients (%)	*p* value
Sex		0.070
Male	56 (54.9%)	
Female	46 (45.1%)	
Age (y)		0.008
< 60	45 (44.1%)	
≥ 60	57 (55.9%)	
Diagnosis		0.503
iCCA	33 (32.4%)	
GBC	14 (13.7%)	
pCCA	55 (53.9%)	
Degree of differentiation		0.078
High differentiation	2 (2%)	
Moderate differentiation	19 (18.6%)	
Poor differentiation	28 (27.5%)	
Unknown	53 (52%)	
Previous treatment		0.447
Yes	25 (24.5%)	
No	77 (75.5%)	
Hepatitis		0.345
No	53 (52%)	
Hepatitis B	49 (48%)	
Hepatitis C	0 (0)	
Child–Pugh classification		0.323
A	52 (51%)	
B	50 (49%)	
ECOG PS		0.013
0	64 (62.7%)	
1	38 (37.3%)	
Jaundice		0.205
Yes	71 (69.6%)	
No	31 (30.4%)	
Extent of disease		0.081
NxM0 + N0M0	41 (40.2%)	
N1M0	53 (52%)	
N2M0 + any M1	8 (7.8%)	
SER_0_		< 0.001
< 1.04	49 (48%)	
≥ 1.04	53 (52%)	
CEA level		< 0.001
< 10 U/mL	74 (72.5%)	
≥ 10 U/mL	25 (24.5%)	
Unknown	3 (2.9%)	
CA 19-9 level		0.001
< 200U/mL	31 (30.4%)	
≥ 200U/mL	67 (65.7%)	
Unknown	4 (3.9%)	
Treatment cycle		0.002
2–4 cycles	54 (52.9%)	
5–6 cycles	48 (47.1%)	
ΔSER		0.031
< 0	36 (35.3%)	
> 0	66 (64.7%)	
Subsequent treatment		0.173
Yes	63 (61.8%)	
No	39 (38.2%)	

iCCA, intrahepatic cholangiocarcinoma; GBC, gallbladder cancer; pCCA, perihilar cholangiocarcinoma; ECOG PS, Eastern Cooperative Oncology Group performance status; SER, signal enhancement ratio; CEA, carcinoembryonic antigen; CA 19-9, carbohydrate antigen 19-9

### Tumor response and survival after HAIC

The follow-up processes were completed on January 23, 2021, with a median follow-up time of 48.6 months. There were 438 cycles of HAIC performed in this study, with a mean of 4.38 cycles. Seventy-seven (75.5%) patients died, and 74 (72.5%) patients had progressed by the date of last follow-up.

Two (2.0%) patients achieved CR, 54 (52.9%) PR, 32 (31.4%) SD, and 14 (13.7%) PD, according to RECIST 1.1 criteria. The objective response rate (ORR) was 54.9%, and the disease control rate (DCR) was 86.3%. After the treatment of HAIC, the median OS and PFS were 14.2 months (range 1.8–83.3 months, 95% confidence interval (CI): 11.106–7.294) and 9.8 months (range 1.5–83.3 months, 95% CI: 7.789–11.811), respectively (Fig. 4A, B).

### Diversity of SER based on different MRI machines

Forty-three (42.2%) patients received CE-MRI scan before the initiation of HAIC with machine 1, 32 (31.4%) with machine 2, and 27 (26.5%) with machine 3. The SER_0_ based on these three different MRI machines was similar (*p* = 0.174), in which SER_0_ based on machine 1 was 1.06 ± 0.51, machine 2 was 1.28 ± 0.59, and machine 3 was 1.09 ± 0.36. Thirty-two (31.4%) patients received CE-MRI scan after 2 cycles of HAIC with machine 1, 31 (30.4%) with machine 2, and 39 (38.2%) with machine 3. The SER_1_ based on these three machines was also similar (*p* = 0.230), in which SER_1_ based on machine 1 was 0.91 ± 0.47, machine 2 was 1.10 ± 0.52, and machine 3 was 0.97 ± 0.42. Additionally, ΔSER was also similar in patients whose CE-MRI was performed with the same machine and patients whose CE-MRI was performed with different machines before and after HAIC (*p* = 0.087). The ΔSER based on the same machine was 0.23 ± 0.63, and the ΔSER based on different machines was 0.04 ± 0.33.

### Relationship between survival, SER_0_ and ΔSER

**Fig. 3:**
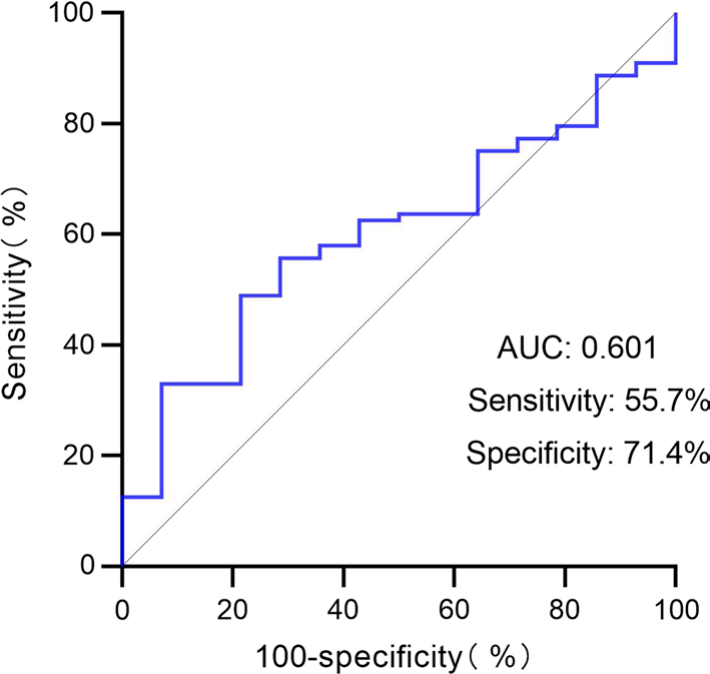
ROC curve of SER_0_. ROC, receiver operating characteristic; AUC, area under the curve

**Fig. 4:**
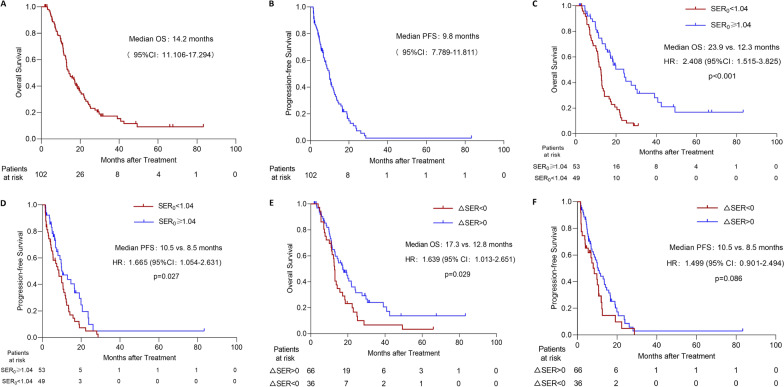
**A**, **B** Cumulative survival curves of hepatic arterial infusion chemotherapy (HAIC) for biliary tract cancers (BTC). **C**, **D** Cumulative survival curves of HAIC for BTC in different SER_0_ groups. **E**, **F** Cumulative survival curves of HAIC for BTC in different ΔSER groups. SER, signal enhancement ratio; OS, overall survival; PFS, progression-free survival; HR, hazard ratio; CI, confident interval

According to the ROC method and Youden Index calculation, the cutoff value of SER_0_ was 1.04 (area under the curve, 0.601; sensitivity, 55.7%; specificity, 71.4%; Fig. 3). Forty-nine (48.0%) patients were included in the SER_0_ < 1.04 group, and fifty-three (52.0%) patients were included in the SER_0_ ≥ 1.04 group. The median OS of patients in the SER_0_ ≥ 1.04 group was 23.9 months (range 1.8–83.3 months, 95% CI: 16.494–31.306), which was significantly longer than that in the SER_0_ < 1.04 group (12.3 months, range 2.9–31.1 months, 95% CI: 10.748–13.852, *p* < 0.001). Similarly, the median PFS of 10.5 months (range 1.6–83.3 months, 95% CI: 6.140–14.860) in the former group was significantly longer than the PFS of 8.5 months (range 1.5–28.7 months, 95% CI: 5.337–11.663, *p* = 0.027, Fig. 4C, D) in the latter group.

**Fig. 5:**
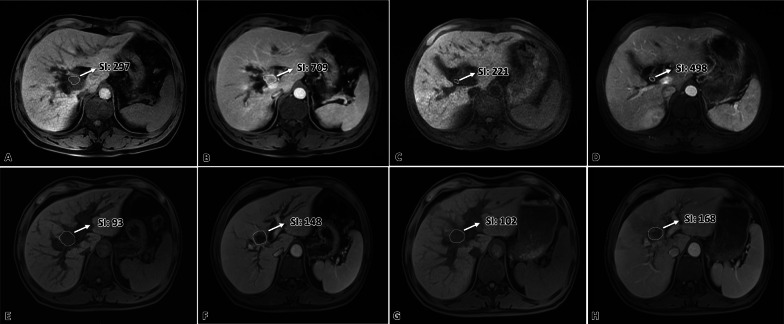
Two typical case demonstrations of perihilar cholangiocarcinoma. **A**–**D** Images from a 47-year-old male patient with pCCA. **A**, **B** The signal enhancement ratio (SER) before the initiation of hepatic arterial infusion chemotherapy (HAIC) was 1.39 [(709–297)/297]. **C**, **D** After two cycles of HAIC, the tumor shrank significantly in size, which corresponded to partial response according to RECIST 1.1. SER of the tumor decreased [SER = (498–221)/221 = 1.25; ΔSER = 1.39–1.25 = 0.14 > 0]. This patient was still alive, and the tumor did not progress by the time of last follow-up, with a survival time more than 83 months. **E**–**H** Images from a 60-year-old male patient with pCCA. **E**, **F** The SER before the initiation of HAIC was 0.59 [(148–93)/93]. **G**, **H** After two cycles of HAIC, the tumor did not change significantly in size, which corresponded to stable disease according to RECIST 1.1. SER of the tumor increased [SER = (168–102)/102 = 0.65; ΔSER = 0.59–0.65 = − 0.06 < 0]. The tumor progressed after 2 months following the initiation of HAIC. Although the subsequent HAIC with another regimen was performed, the patient died 5.4 months after the initiation of HAIC

**Fig. 6:**
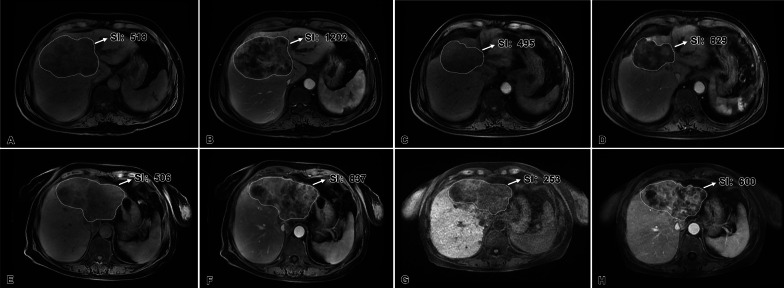
Two typical case demonstrations of intrahepatic cholangiocarcinoma. **A**–**D** Images from a 66-year-old male patient with iCCA. **A**, **B** The signal enhancement ratio (SER) before the initiation of hepatic arterial infusion chemotherapy (HAIC) was 1.32 [(1202–518)/518]. **C**, **D** After two cycles of HAIC, the tumor shrank significantly in size, which corresponded to partial response according to RECIST 1.1. SER of the tumor decreased [SER = (829–495)/495 = 0.67; ΔSER = 1.32–0.67 = 0.65 > 0]. The tumor progressed after 10 months following the initiation of HAIC. Without subsequent treatment after progression, the patient died after 11.2 months of the initiation of HAIC. (E–H) Images from a 76-year-old female patient with iCCA. **E**, **F** The SER before the initiation of HAIC was 0.65 [(837–506)/506]. **G**, **H** After two cycles of HAIC, the tumor did not change significantly in size, which corresponded to stable disease according to RECIST 1.1. SER of the tumor increased [SER = (600–253)/253 = 1.37; ΔSER = 0.65–1.37 = − 0.72 < 0]. The tumor progressed after 4 months following the initiation of HAIC, and subsequent HAIC with another regimen was performed. The patient died 13.7 months after the initiation of HAIC with 3cir-OFF regimen

All patients were divided into two groups based on ΔSER. There were 66 (64.7%) patients in the ΔSER > 0 group, and 36 (35.3%) in the ΔSER < 0 group. Although the median PFS in the ΔSER > 0 and ΔSER < 0 groups was similar (10.5 vs. 8.5 months, *p* = 0.086), the median OS of 17.3 months (range 1.8–83.3 months, 95% CI: 12.359–22.241) in the former group was significantly longer than the 12.8 months [range 4.0–66.0 months, 95% CI: 12.134–13.466, *p* = 0.029, Figs. 4E, F, 5, 6) in the latter group.

### Predictors of survival after HAIC

Sex (*p* = 0.070), age (*p* = 0.008), degree of differentiation (*p* = 0.078), extent of disease (*p* = 0.081), ECOG PS (*p* = 0.013), SER_0_ (*p* < 0.001), CEA level (*p* < 0.001), CA 19-9 level (*p* = 0.001), treatment cycle (*p* = 0.002), and ΔSER (*p* = 0.031) were identified as risk factors via univariate analysis. In the multivariate analysis, SER_0_ (*p* = 0.029) and treatment cycle (*p* = 0.002) were confirmed as independent risk factors related to survival (Table 2).

**Table 2:** Multivariate Cox regression analysis of factors related to survival

Variates	*p* value	HR (95% CI)
Sex	0.489	1.358 (0.571, 3.230)
Age	0.438	0.700 (0.284, 1.726)
Degree of differentiation		
High/moderate	0.353	0.324 (0.030, 3.502)
High/poor	0.052	0.380 (0.143, 1.010)
Extent of disease		
NxM0 + N0M0/N1M0	0.451	0.606 (0.165, 2.228)
NxM0 + N0M0/N2M0 + any M1	0.404	0.587 (0.168, 2.051)
ECOG PS	0.465	0.725 (0.306, 1.719)
SER_0_	0.029	2.686 (1.109, 6.505)
CEA level	0.055	0.472 (0.219, 1.017)
CA 19-9 level	0.194	0.576 (0.251, 1.324)
Treatment cycle	0.002	3.669 (1.611, 8.353)
ΔSER	0.779	1.137 (0.463, 2.793)

HR, hazard ratio; CI, confident interval; ECOG PS, Eastern Cooperative Oncology Group performance status; SER, signal enhancement ratio; CEA, carcinoembryonic antigen; CA 19-9, carbohydrate antigen 19-9

## Discussion

This large 10-year cohort retrospective study demonstrated the prediction value of signal enhancement ratio from CE-MRI as a biomarker of survival after hepatic arterial infusion chemotherapy. In this study, median OS and PFS in SER before HAIC (SER_0_) > 1.04 group were significantly longer than those in the SER_0_ < 1.04 group (median OS: 23.9 vs. 12.3 months, *p* < 0.001; median PFS: 10.5 vs. 8.5 months, *p* = 0.027). Further, the median OS of patients in the early change of SER (ΔSER) > 0 group was better than that in the ΔSER < 0 group (17.3 vs. 12.8 months, *p* = 0.029). Additionally, SER_0_ was an independent risk factor of survival according to the multivariate analysis.

Several potential biomarkers have currently been identified as being associated with the OS of BTC, such as CA 19-9, serum IL-6, and neutrophil/lymphocyte ratio [17–21]. However, the results of studies that focused on these biomarkers were contradictory. Additionally, the use of these biomarkers in the clinical setting remains controversial due to poor specificity, especially CA 19-9, which may be elevated in other conditions such as diabetes [19, 22, 23]. The results of this study suggest that SER may be a reliable biomarker for prediction of survival after HAIC treatment of patients with advanced BTC.

The OS and PFS of patients in the SER_0_ > 1.04 group was longer than those in the SER_0_ < 1.04 group. This may be due to the following reasons. First, tumor angiogenesis has been proved to be necessary for the growth of tumors [24, 25], and the enhancement of tumor in CE-MRI is partially associated with vascularity and high permeability of the tumor [26, 27]. Therefore, in this study, the greater SER_0_, the more vessels or the higher vessel permeability of the BTC before the initiation of HAIC. Second, the adequate delivery and transport of chemotherapeutics to the tumor, which depends in part on the tumor vessels and their permeability, would increase the efficacy of chemotherapy [28]. Third, HAIC could increase the local concentration of chemotherapeutics by infusing the chemotherapeutics into the vessels supplying the tumor directly. Thus, more chemotherapeutics would be infused into the tumor in the SER_0_ > 1.04 group, resulting in better survival. Furthermore, it is well known that the differentiation of a tumor can influence prognosis. Some published studies had found better enhancement of iCCA was associated with well differentiation of tumors [29, 30]. Jeong et al. also found that mass-forming iCCA with well differentiation tended to present with greater enhancement in HBP [16]. Thus, SER_0_ > 1.04 might also correlate to the well differentiation of tumor.

Enhancement is considered one factor in the evaluation of the treatment response according to the modified RECIST for HCC [31]. However, in 2017, Chen et al. found that retinoblastoma with decreased activation after the treatment of HAIC presented with lower enhancement in MRI [32]. Another recently published study also proved that the enhancement of tumors decreased after the effective chemotherapy [33]. Similar results were also observed in other tumor types, such as breast cancer after neoadjuvant chemotherapy and locally advanced pancreatic cancer after high-intensity focused ultrasound [34, 35]. Therefore, both absence and decrease in tumor enhancement could reflect response after effective treatment. HAIC using the 3cir-OFF regimen has been proved to be an effective and safe treatment for BTC [7, 8]. In this study, the median OS and PFS were 14.2 and 9.8 months, respectively, although 24.5% of patients with prior treatment failed. The survival benefits of HAIC with the 3cir-OFF regimen were superior to results achieved in treatment-naïve BTC patients treated with systemic chemotherapies such as gemcitabine plus cisplatin, XELOX, GEMOX, S-1 along, gemcitabine plus S-1, or FOLFIRI (median OS: 11.7, 10.4, 10.6, 9.4, 11.6, and 6.6 months, respectively) [4, 5, 36–38]. Further, ΔSER > 0 showed that the enhancement of BTC decreased after the treatment of HAIC in this study. The median OS in the ΔSER > 0 group was 17.3 months, which was significantly longer than that in the ΔSER < 0 group with a median OS of 12.8 months (*p* = 0.029). These results are consistent with the fact that the decrease in tumor enhancement reflected the response after effective treatment.

This study has some limitation. First, this was a retrospectively study with only 102 patients enrolled. Second, the number of patients with GBC, iCCA, or pCCA varied greatly, and the number of patients with high, moderate, or poor degree of differentiation of BTC varied greatly in this study. Third, most patients enrolled in this study were diagnosed with BTC by cytopathology rather than histopathology, which limited the further investigation of the relationship between SER and the expression of certain molecules or proteins. Finally, some biases were inevitable when the ROI was drawn, although the ROI included as more substantial components as possible.

In conclusion, signal enhancement ratio of contrast-enhanced MRI is an independent biomarker for prediction of survival after hepatic arterial infusion chemotherapy of advanced biliary tract cancers. The early change of SER (ΔSER) was also related to longer survival. Therefore, a prospective trial is needed to verify the results in this study.

## Data Availability

The data and material supporting the results reported in this study can be found at Peking University Cancer Hospital.

## Abbreviations

BTC: Biliary tract cancers
CA 19-9: Carbohydrate antigen 19-9
CEA: Carcinoembryonic agent
CE-MRI: Contrast-enhanced MRI
GBC: Gallbladder cancer
HAIC: Hepatic arterial infusion chemotherapy
iCCA: Intrahepatic cholangiocarcinoma
OS: Overall survival
pCCA: Perihilar cholangiocarcinoma
PFS: Progression-free survival
SER: Signal enhancement ratio
SI: Signal intensity
